# Effects of Dietary Yeast β-Glucan Supplementation on Meat Quality, Antioxidant Capacity and Gut Microbiota of Finishing Pigs

**DOI:** 10.3390/antiox11071340

**Published:** 2022-07-08

**Authors:** Linjuan He, Jianxin Guo, Yubo Wang, Lu Wang, Doudou Xu, Enfa Yan, Xin Zhang, Jingdong Yin

**Affiliations:** State Key Laboratory of Animal Nutrition, College of Animal Science and Technology, China Agricultural University, Beijing 100193, China; sy20193040690@cau.edu.cn (L.H.); s20203040611@cau.edu.cn (J.G.); wangyb1994@cau.edu.cn (Y.W.); bs20193040399@cau.edu.cn (L.W.); bs20183040388@cau.edu.cn (D.X.); b20203040325@cau.edu.cn (E.Y.); yinjd@cau.edu.cn (J.Y.)

**Keywords:** yeast β-glucan, pork quality, glycolytic potential, antioxidant capacity, gut microbiota

## Abstract

Yeast β-glucan is a natural antioxidant and has been reported to improve growth performance of piglets, but its application in improving pork quality is limited. This study investigated the effects of dietary yeast β-glucan supplementation on meat quality, antioxidant capacity and gut microbiota of finishing pigs. In a 40-day experiment, ninety finishing pigs (Duroc × Landrace × Yorkshire, 70.47 ± 0.04 kg) were randomly allocated into five treatments including a basal diet supplemented with 0, 50, 100, 200 and 400 mg/kg yeast β-glucan. Results showed that yeast β-glucan significantly increased pH_45 min_ (linear and quadratic, *p* < 0.01) and a*_45 min_ (linear, *p* < 0.05), and reduced cooking loss (linear, *p* < 0.05) and drip loss (quadratic, *p* < 0.05) of meat in finishing pigs. Importantly, the 200 mg/kg group exhibited the highest values of pH_45 min_ (*p* < 0.01) and the lowest values of drip loss (*p* < 0.05), accompanied by a decreased lactate content (*p* < 0.05) and glycolytic potential (*p* < 0.05). Dietary supplementation of 200 mg/kg yeast β-glucan markedly increased catalase (CAT) (*p* < 0.05), superoxide dismutase (SOD) (*p* < 0.05) and total antioxidant capacity (T-AOC) (*p* < 0.01) activities in skeletal muscle. Moreover, *WPS-2* abundance was decreased significantly in colonic digesta by 200 mg/kg yeast β-glucan and exhibited a positive association with muscle lactate content and drip loss. Together, dietary 200 mg/kg yeast β-glucan supplementation effectively improved pH value and the water-holding capacity of fresh meat through reducing muscle postmortem glycolysis, increasing antioxidant capacity and altering the gut microbiota composition of finishing pigs.

## 1. Introduction

Pork is one of the most commonly consumed meat products worldwide. Along with the intense pursuit of growth and lean meat percentage of pigs, pork quality has deteriorated as shown by the great drip loss and poor appearance [1]. Briefly, greater drip loss contributes to a reduced water holding capacity (WHC) and thus negatively affects the meat yield [2]. Lower postmortem pH values also account for the lower WHC, paler color of pork and poor processing yield [3]. It should be noted that muscle postmortem glycolysis and oxidative status are widely considered as important indicators of meat quality [4,5]. Briefly, postmortem extended glycolysis drives hydrogen and lactate accumulation in muscle and results in a lower ultimate pH value [6,7] and an elevation in drip loss and L^*^ values [8]. Peroxidation is another negative factor that has an adverse impact on meat quality. The primary and secondary metabolites of oxidative reactions in muscles, such as short-chain aldehydes and ketones, contribute to the loss of meat color and the reduction of nutritional value [9]. Antioxidants can maintain the oxidative stability of meat [10] and mitigate glycolysis through a decreasing accumulation of cellular reactive oxygen species (ROS) [11] or stimulating antioxidant enzymes, thus effectively improving meat quality [12].

Yeast β-glucan is a kind of functional polysaccharide widely spreading in the cell wall of yeast. Unlike β-glucan derived from bacteria and cereal, yeast β-glucan has frequent β-1,3-D-glucose side chains at β-1,6 branching points, which is easily recognized and accepted by the immune system [13]. Yeast β-glucan has various biological functions such as promotion on immune function [14,15], antistress [16] and gut health [17]. Importantly, the antioxidant capacity of yeast β-glucan has been corroborated due to its susceptibility to free radical degradation [18]. Multiple reports have revealed that as one natural antioxidant, yeast β-glucan could inhibit methotrexate-induced leukocyte apoptosis [16], reduce plasma lipid peroxidation induced by haloperidol [19] and alleviate oxidative stress of macrophages via the Dectin-1/Nrf2/HO-1 signaling pathway [20]. In husbandry animal production, yeast β-glucan has also been used to reduce diarrhea and inflammation [21] and enhance daily weight gain of piglets [22]. In view of the foregoing, yeast β-glucan as a natural antioxidant may be of great interest for improving pork quality. Although the ameliorative effects of β-glucan derived from oat, algae and bacteria on pH values and drip loss have been revealed in previous studies [23,24], the contribution of dietary yeast β-glucan supplementation to pork quality has not been reported yet.

Emerging studies have focused on the interactions between gut microbiota and skeletal muscle metabolism [25,26]. Of particular interest was the positive correlation of intramuscular fat (IMF) content and the ratio of *Firmicutes* to *Bacteroidetes* evidenced by fecal microbiota transplantation [27]. The causal role of *Prevotella copri* in the fat deposition of pigs was also revealed previously [28]. Similarly, as key metabolites produced after the bacterial fermentation of nondigestible carbohydrate, short-chain fatty acids (SCFAs) were evidenced to induce the slow-switch myofiber formation [29] and reduce the drip loss of pork [30], implying that gut microbiota is an important determinant of meat quality traits. The impact of yeast β-glucan on porcine gut microbiota has been reported in the pre-weaning period [31], however, whether it could regulate skeletal muscle metabolism and pork quality through altering gut microbiota remains mysterious.

Consequently, the objective of this study is to evaluate the effects of dietary yeast β-glucan supplementation on the carcass characteristics, meat quality, antioxidant capacity as well as gut microbiota of finishing pigs. This study provides novel knowledge concerning yeast β-glucan as a promising natural antioxidant to improve the WHC of fresh meat.

## 2. Materials and Methods

### 2.1. Animals and Experimental Design

A total of ninety healthy Duroc × Landrace × Yorkshire castrated pigs with an average body weight (BW) of 70.47 ± 0.04 kg were selected and allocated into five groups according to their initial BW. Eighteen replicates were involved in each group. A corn–soybean-based basal diet was formulated to meet the NRC (2012) nutrient requirements for pigs of 75–100 kg BW. Yeast β-glucan from *Saccharomyces cerevisiae* (effective content 95%, Biorigin, Brazil) at levels of 0, 50, 100, 200 and 400 mg/kg was supplemented to the basal diet, respectively. The chosen dosage was in reference to studies conducted in piglets [32] with some modifications. All pigs had free access to feed and clean drinking-water for 40 d. The ingredient composition and nutrient levels of basal diet are presented in Table 1.

### 2.2. Sample Collection

At the end of the experiment, pigs close to the average final BW of each group (*n* = 8) were selected and fasted for 12 h. Blood samples were collected from the precaval vein, and plasma was separated and stored at −20 °C for further analysis. These pigs were transported to a local abattoir and humanly slaughtered by electrical stunning after at least 8 h rest. The *Longissimus thoracis* (LT) muscle from the right-side carcass at the 10th rib was sampled and frozen in −20 °C for fatty acid composition analysis or stored at −80 °C for RNA extraction and chemical analysis. In addition, fresh digesta within the distal colon were collected and stored at −80 °C for the measurement of SCFAs contents and microbiota composition.

### 2.3. Carcass Traits

Hot carcass weight was recorded immediately, and dressing percentage was calculated dividing the hot carcass weight by the final BW. Carcass length, backfat thickness and loin eye area were measured and calculated at the slaughter spot by Chinese Guidelines on Performance Measurement Technology and Regulations for Pigs. Briefly, carcass length was measured as the distance between the anterior edge of pubic symphysis to the midpoint of the first rib and sternum. The values of backfat thickness opposite the thickest shoulder, the last rib, the 6th to 7th rib, the 10th rib, and the last lumbar vertebra, as well as the longitudinal dorsal muscles at the 10th rib in a vertical direction were used to measure the loin eye area according to the following equation: loin eye area (cm^2^) = loin eye height (cm) × loin eye width (cm) × 0.7. The fat-free lean index was estimated as follows: fat-free lean index = 50.767 + [0.035 × hot carcass weight (lb)] – [8.979 × the last rib fat thickness (in.)] (NPPC, Des Moines, Iowa, 1994).

### 2.4. Meat Quality

The LT muscle between the 10th and 12th ribs was subjected to a meat quality evaluation. Subjective color and marbling score on the LT muscle were evaluated according to the Official Color Standards (National Pork Producers Council, Des Moines, IA, USA), a score of 1.0 is very pale, white and a score of 6.0 is dark purplish red (NPPC 1999). At 45 min postmortem, the meat color, including L* (lightness), a* (redness), and b* (yellowness) values, was measured by a colorimeter (Minolta ChromaMeter, CR-410, Konica Minolta, Osaka, Japan) according to the standard method of the CIELab system. The colorimeter was calibrated against a white tile in accordance with the manufacturer’s manual before measurement. Meanwhile, the initial muscle’s pH_45 min_ was measured with a SPK pH meter (pH-star, DK2730, Herlev, Denmark), which was calibrating with pH 4.6 and 7.0 buffers. Moreover, the pH_24 h_ value was recorded and calculated at 24 h postmortem in a 4 °C chilling room. Drip loss (%) was calculated as fluid loss after storage of the LT muscle sample in plastic bags at 4 °C for 24 h and was calculated by the equation: drip loss (%) = [(initial weight – final weight) / initial weight] × 100. In addition, approximately 100 g of each meat sample was weighed, sealed in a plastic bag and heated in a constant temperature water bath with 75 °C water for 30 min in one cooking batch, and then residual moisture was removed from the meat sample, and the latter was cooled to room temperature and reweighed to calculate cooking loss. Shear force was determined as described in our previous study using a digital-display muscle tenderness meter (C-LM3B, Tenovo, Harbin, China) with a speed of 300 mm/min [33]. The mean value for 10 subsamples of each sample was recorded.

About 50 g of the LT muscle sample of each pig was cut into thin slices (2–3 mm) and weighed in aluminum boxes. The aluminum boxes were put into a freeze dryer (Freezone 4.5™, Labconco Corp., Kansas City, MO, USA) for 48 h with a temperature of −60 °C, and then reweighed. The percentage of moisture was determined by calculating the difference between the initial and the dried muscle sample weight. Lyophilized muscle was subsequently ground into powder and subsequently analyzed for crude protein content, IMF content and fatty acid composition. The crude protein content was measured according to Association of Analytical Chemists methods (AOAC, Rockville, MD, USA, 2007). The IMF content was analyzed by Soxhlet petroleum-ether extraction (Budwi Extraction System B-11; Budwi, Lausanne, Switzerland) as previously described [34]. IMF content was then converted to the weight percentage of fresh meat weight.

### 2.5. Texture Characteristics

The LT muscle samples were heated in water bath with 75 °C water for 30 min. After cooling to room temperature, samples were cut into uniform cubes of approximately 1 cm^3^. Texture parameters including adhesiveness, springiness, cohesiveness, gumminess, chewiness, hardness and shear force were measured by a texture analyzer (TMS-Touch, Food Technology Corp., Sterling, VA, USA) with a p 0.5 cylindrical probe of ebonite. Parameters were as follows: pretest speed, 0.5 mm/s; test speed, 1.0 mm/s; post-test 1.0 mm/s; trigger type, auto −5 g; and strain, 50%.

### 2.6. Muscle Fatty Acid Profiles

Lyophilized muscle samples (150–200 mg) were extracted using 4 mL acetyl/anhydrous methanol (1/10, V/V) solution, treated with 1 mL n-hexane and 1 mL internal standard FA solution (1 mg/mL C11:0), and then hydrolyzed for 2.5 h in an 80 °C water bath. After cooling to room temperature, 5 mL of 7% K_2_CO_3_ was added. The samples were mixed and centrifuged for 3 min at 900 rpm/min. The supernatant was subjected to gas chromatography analysis (Agilent Technologies Inc, Santa Clara, CA, USA). Fatty acid profile was shown as a percentage of fresh muscle tissue.

### 2.7. Plasma and Skeletal Muscle Biochemical Parameters

The content of albumin (ALB) and total protein (TP) in plasma was determined by the automatic biochemistry analyzer (Beckman BS-420; Beckman Coulter Inc, Brea, CA USA), and the difference between TP minus ALB was the globulin (GLB) contents. Cortisol contents in plasma samples were measured using ELISA kits (Nanjing Angle Gene Bioengineering Company, Nanjing, China) following the manufacturer’s instructions.

To determine the glycolytic potential of skeletal muscle, the glucose, glucose-6-phosphate, glycogen and lactate contents in LT samples were measured using commercial kits (Nanjing Jiancheng Bioengineering Company, Nanjing, China) according to the manufacturer’s instructions. Glycolytic potential (μmol·g^−1^) was calculated as follows: glycolytic potential = 2 (glycogen content + glucose content + glucose-6-phosphate content) + lactate content [35]. The activities of pyruvate kinase M1 (PKM1), lactate dehydrogenase (LDH), glyceraldehyde-3-phosphate dehydrogenase (GAPDH) and phosphoglycerate kinase 1 (PGK1) in the LT muscle were also measured using ELISA kits (Nanjing Angle Gene Bioengineering Company, Nanjing, China). Furthermore, the activities of SOD, T-AOC, CAT, glutathione peroxidase (GSH-Px) and the content of malondialdehyde (MDA) in LT muscle sample from each pig were determined using commercial kits (Nanjing Jiancheng Bioengineering Institute, Nanjing, China). The level of reactive oxygen species (ROS) in the LT muscle were also measured using commercial kits (Beijing Huaying Bioengineering Company, Beijing, China).

### 2.8. RNA Extraction and qRT-PCR Analysis

Total RNA was extracted from LT muscle using RNAiso Plus (Takara, Beijing, China) and then reverse-transcribed into cDNA by a PrimeScript™ RT reagent kit (Takara, Beijing, China) according to the manufacturer’s protocol. SYBR-Green-based quantitative PCR was conducted in a qTOWER 2.2 thermocycler (Analytik Jena AG, Jena, Thuringia, Germany). Moreover, 18S RNA was used as the internal control to normalize the expression of target genes. The primers used in this study were listed in Table S1. Relative gene expression was calculated by 2^−ΔΔCt^ method [36].

### 2.9. SCFAs Concentration in Colonic Digesta

The concentration of SCFAs in colonic digesta was measured as previously described [37]. Briefly, 1 g of colonic digesta samples were weighed and added to 8 mL of deionized water, dissolved, homogenized and then centrifuged at 5000× *g* for 10 min. The supernatant was diluted 50 times and filtered through a 0.22 μm filter (Millipore, Bedford, UK). Extracted sample solution (25 μL) was kept in a 2 mL screw-cap vial, and then subjected to SCFAs concentration determination with an ion chromatography system (Thermo Fisher Scientific, Wilmington, DE, USA).

### 2.10. DNA Extraction and PCR Amplification

Microbial DNA from the colonic contents was extracted using a commercial kit (Omega Bio-Tek, Inc., Norcross, GA, USA) according to the manufacturer’s protocol. Final DNA concentration and purification were determined using the NanoDrop 2000 UV–vis spectrophotometer (Thermo Fisher Scientific, Wilmington, DE, USA) and DNA quality was assessed by 1% agarose gel electrophoresis. The V3–V4 hypervariable regions of the bacterial 16S rRNA gene were amplified with primers 338 F (5′-ACTCCTACGGGAGGCAGCAG-3′) and 806 R (5′- GGACTACHVGGGTWTCTAAT-3′) using a thermocycler (GeneAmp 9700, ABI, Waltham, MA, USA). The PCR products were extracted using 2% agarose gel, purified using the AxyPrep DNA Gel Extraction Kit (Axygen Biosciences, Union City, CA, USA) and quantified using QuantiFluorTM-ST (Promega, Madison, WI, USA) following the manufacturer’s instructions.

### 2.11. DNA Illumina miSeq and Sequence Data Processing

Purified amplicons were pooled in equimolar concentrations and paired-end sequenced (2 × 300) on an Illumina MiSeq platform (Illumina, Inc., San Diego, CA, USA) according to the standard protocol by Majorbio Bio-Pharm Technology, Co., Ltd., (Shanghai, China). The raw reads were deposited into the NCBI Sequence Read Archive (SRA) database (Accession Number: SRP218537). The quality of the raw fastq files were filtered, operational taxonomic units (ASVs) were clustered, and the taxonomy of each 16S rRNA gene sequence was analyzed as previously described [38].

### 2.12. Statistical Analysis

All data in the five groups are presented as means ± SEMs and were analyzed by the one-way ANOVA model followed by Tukey’s multiple-range tests using SAS 8.2 software (SAS Inst. Inc., Cary, NC, USA). Linear and quadratic regression analyses were also performed to evaluate the dose-response of yeast β-glucan. A two-tailed Student’s *t*-test or a one-way ANOVA was used to determine the effects of 200 mg/kg yeast β-glucan supplementation on mRNA expression levels, enzyme activities, SCFAs concentration and α-diversity. The bacterial diversity with standardized ASV reads was analyzed using R-software (version 3.6.3); the bacterial abundances at the phylum and genus levels are presented in bar plots. The bacterial community in the colonic digesta was analyzed using the Wilcoxon rank-sum test. The Pearson correlation coefficient was used for correlation analysis. Differences were regarded as significant at *p* < 0.05, and 0.05 ≤ *p* ≤ 0.10 was considered to have a trend.

## 3. Results

### 3.1. Immunological Stress

As shown in Table 2, compared to control, dietary supplementation of yeast β-glucan significantly increased plasma concentrations of ALB (linear, *p* < 0.01; quadratic, *p =* 0.05) and GLB (quadratic, *p* < 0.01), whereas it reduced the ratio of ALB to GLB (A/G) (linear, *p* < 0.05; quadratic, *p* < 0.01). Additionally, dietary supplementation of yeast β-glucan significantly decreased the plasma concentration of cortisol (quadratic, *p* < 0.05). Compared to the control, 200 mg/kg yeast β-glucan supplementation significantly increased the contents of ALB and GLB and decreased the values of A/G and plasma cortisol contents (*p* < 0.05).

### 3.2. Carcass Traits and Meat Quality

As shown in Table 3, dietary supplementation of yeast β-glucan did not affect most carcass characteristics, such as carcass length, dressing percentage, loin eye area and average backfat thickness in finishing pigs (*p* > 0.05). The last rib fat thickness and 10th rib fat thickness were significantly increased by yeast β-glucan in a quadratic or liner manner (*p* < 0.05). Compared to the control, dietary 100 mg/kg yeast β-glucan supplementation significantly decreased the fat-free lean index of finishing pigs (*p* < 0.05).

Importantly, in terms of meat quality traits, dietary supplementation of yeast β-glucan significantly increased pH_45 min_ (linear and quadratic, *p* < 0.01), a*_45 min_ (linear, *p* < 0.05) and the marbling score (quadratic, *p* < 0.05) of the meat (Table 4). Meanwhile, the L*_45 min_, cooking loss (linear, *p* < 0.05) and drip loss (quadratic, *p* < 0.05) decreased after yeast β-glucan supplementation (Table 4). In particular, dietary supplementation of 200 mg/kg yeast β-glucan markedly increased pH_45 min_ and decreased drip loss compared to the control (*p* < 0.05) (Table 4), suggesting an improvement in pork quality by 200 mg/kg yeast β-glucan supplementation. Other meat quality traits showed no differences among dietary treatments, including pH_24 h_, b*_45 min_, shear force and muscular contents of moisture, crude protein and IMF.

### 3.3. Texture Profile Analysis and Fatty Acid Composition of Fresh Meat

As illustrated in Table S2, dietary supplementation of yeast β-glucan showed little effects on texture characteristics of pork in terms of hardness, adhesiveness, springiness, gumminess and chewiness. Regarding fatty acid profiles in the LT muscle, dietary yeast β-glucan supplementation increased the proportion of stearic acid (C18:0) (quadratic, *p* < 0.05), while no changes were observed in proportions of other fatty acids (Table S3).

### 3.4. Muscle Glycolytic Potential

**Figure 1:**
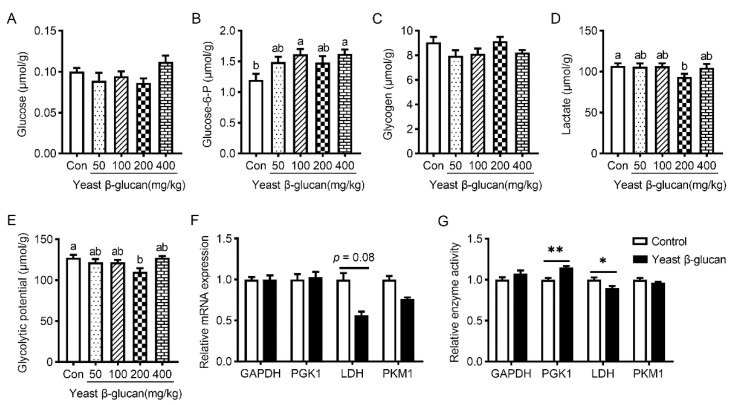
Effects of dietary yeast β-glucan supplementation on indices relating glycolytic potential of the *Longissimus thoracis* muscle in finishing pigs. (**A**–**D**) The contents of glucose, glucose-6-P, glycogen and lactate in the skeletal muscle. (**E**) LT muscle glycolytic of finishing pigs. (**F**) mRNA levels of *PKM1*, *GAPDH*, *LDH* and *PGK1* in the skeletal muscle. (**G**) Activities of pyruvate kinase M1 (PKM1), glyceraldehyde-3-phosphate dehydrogenase (GAPDH), lactate dehydrogenase (LDH) and phosphoglycerate kinase 1 (PGK1) in the skeletal muscle. Yeast β-glucan, dietary supplementation of 200 mg/kg yeast β-glucan. Data are expressed as the means ± SEMs (*n* = 8). Values with different superscript letters are significantly different (*p* < 0.05), and a statistical analysis between two groups was conducted using a two-tailed Student’s *t*-test. * *p* < 0.05, ** *p* < 0.01.

Compared to the control, dietary 200 mg/kg yeast β-glucan supplementation did not affect contents of glucose, glucose-6-phosphate and glycogen (*p* > 0.05) (Figure 1A–C), but significantly decreased the lactate concentration and glycolytic potential of the LT muscle (*p* < 0.05) (Figure 1D,E). Congruently, dietary 200 mg/kg yeast β-glucan supplementation tended to decrease the mRNA abundance of *LDH* in the LT muscle (*p* = 0.08) (Figure 1F), while yeast β-glucan also markedly decreased LDH activity (*p* < 0.05) and increased PGK1 activity (*p* < 0.01) (Figure 1G). No differences were observed in expression levels of *GAPDH*, *PGK1* and *PKM1* upon yeast β-glucan supplementation, nor in activities of GAPDH and PKM1 (*p* > 0.05) (Figure 1F,G). Considering the positive effects of 200 mg/kg yeast β-glucan supplementation on meat quality, we focused on the effects of yeast β-glucan on the muscle fiber characteristics, antioxidant capacity and gut microbiota composition of finishing pigs by employing the supplementation of 200 mg/kg yeast β-glucan in diets, named yeast β-glucan treatment in the subsequent analysis.

### 3.5. Muscle Fiber Characteristics

The expression levels of muscle-fiber-type-related genes of the LT muscle were examined. Dietary 200 mg/kg yeast β-glucan supplementation significantly increased the *MyHC IIx* mRNA expression levels, but had no effects on *MyHC I, MyHC IIa* and *MyHC IIb* mRNA levels (Figure S1).

### 3.6. Muscle Antioxidant Activities

**Figure 2:**
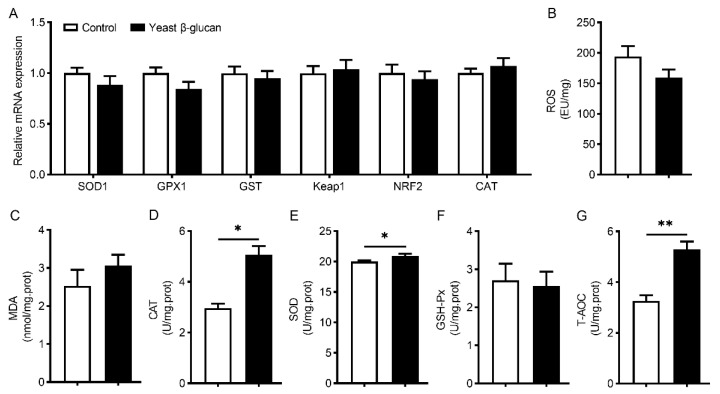
Effects of dietary yeast β-glucan supplementation on mRNA expression levels of antioxidant related genes, reactive oxygen species (ROS) level, malondialdehyde (MDA) content and antioxidant enzyme activities in the *Longissimus thoracis* muscle of finishing pigs. (**A**) mRNA expression levels of *SOD1*, *GPX1*, *GST*, *Keap1*, *NRF2* and *CAT*. (**B**) ROS levels, (**C**) MDA content and (**D**–**G**) activities of catalase (CAT), superoxide dismutase (SOD), glutathione peroxidase (GSH-Px) and total antioxidant capacity (T-AOC) in LT muscle of finishing pigs. Yeast β-glucan, dietary supplementation of 200 mg/kg yeast β-glucan. Data are expressed as the means ± SEMs (*n* = 8). The statistical analysis was conducted using a two-tailed Student’s *t*-test. * *p* < 0.05, ** *p* < 0.01.

As shown in Figure 2, there were no significant differences in mRNA levels of *SOD1, GPX1, GST, Keap1, NRF2* and *CAT* (Figure 2A), levels of reactive oxygen species (ROS) (Figure 2B), content of MDA (Figure 2C) and activities of GSH-Px (Figure 2F) in LT muscle between control and 200 mg/kg yeast β-glucan groups (*p* > 0.05). However, 200 mg/kg yeast β-glucan supplementation significantly increased CAT (*p* < 0.05), SOD (*p* < 0.05) and T-AOC (*p* < 0.01) activities in LT muscle (Figure 2D,E,G).

### 3.7. Responses of Colonic Microbiota to Dietary Yeast β-Glucan Supplementation

**Figure 3:**
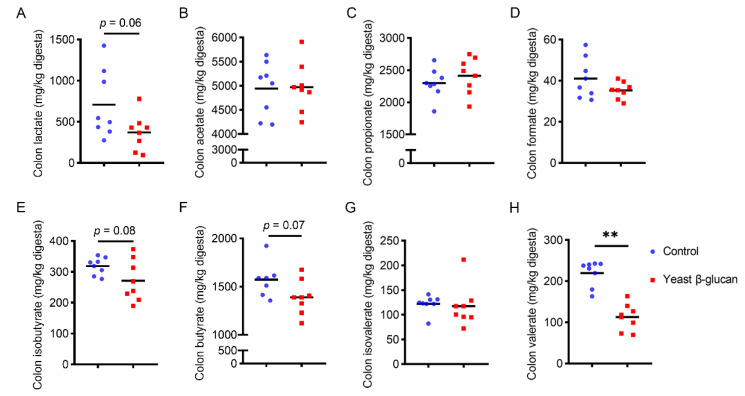
Effects of dietary yeast β-glucan supplementation on the concentrations of SCFAs in colonic digesta in finishing pigs. Contents of (**A**) lactate, (**B**) acetate, (**C**) propionate, (**D**) formate, (**E**) isobutyrate, (**F**) butyrate, (**G**) isovalerate and (**H**) valerate in colonic digesta of finishing pigs. Yeast β-glucan, dietary supplementation of 200 mg/kg yeast β-glucan. Data are expressed as the means ± SEMs (*n* = 8). The statistical analysis was conducted using a two-tailed Student’s *t*-test. ** *p* < 0.01.

Yeast β-glucan tended to decrease the concentration of lactate, isobutyrate and butyrate in the colon digesta (0.05 < *p* < 0.1) (Figure 3A,E,F). Moreover, the colonic digesta valerate concentration was markedly decreased by 200 mg/kg yeast β-glucan supplementation compared with that in control group (*p* < 0.01) (Figure 3H).

**Figure 4:**
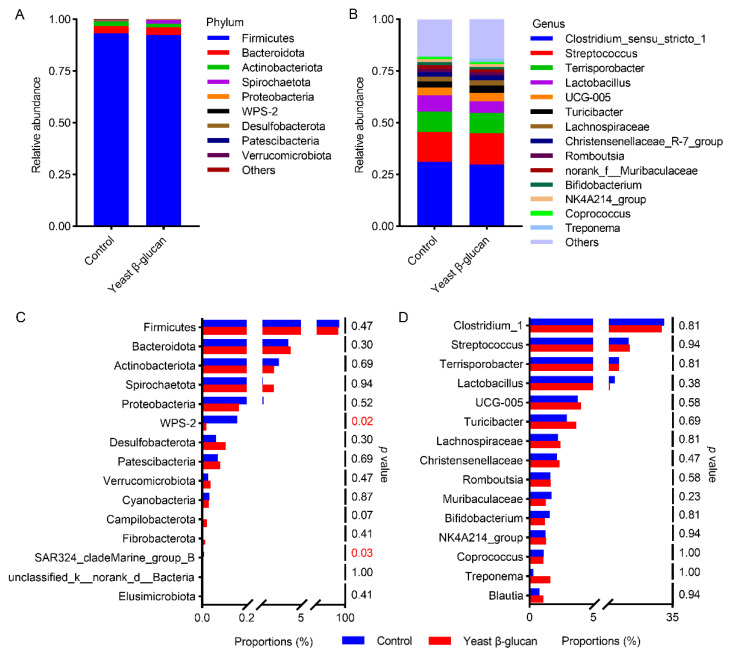
Effects of dietary yeast β-glucan supplementation on the composition of microbial community and the differential taxa in finishing pigs. Relative abundance of (**A**) top 9 intestinal flora at the phylum level and (**B**) top 14 intestinal flora at the genus level in the colon digesta of pigs in the control and 200 mg/kg yeast β-glucan groups. (**C**,**D**) The bar graph shows the most differential taxa between treatment at the phylum and genus level. Yeast β-glucan, dietary supplementation of 200 mg/kg yeast β-glucan. Statistical analyses were performed using a Kruskal–Wallis test with *p* value adjustment using FDR correction. The significance between community structure was evaluated by PERMANOVA (*n* = 6).

Moreover, the α diversity shown by the diversity index of ASV level was not impacted by yeast β-glucan (Figure S2). At the phylum level, *Firmicutes*, *Bacteroidota*, *Actinobacteriota* and *Spirochaetota* were predominant (Figure 4A). At the genus level, *Clostridium_sensu_stricto_1* dominated, accounting for over 30% of the relative abundance followed by *Streptcoccus*, *Terrisporobacter* and *Lactobacillus* (Figure 4B). We further performed a Wilcoxon rank-sum test using phylum and genus, respectively, and identified several phyla closely associated with yeast β-glucan supplementation, including *WPS-2* and *SAR324_cladeMarine_group_B* (Figure 4C,D). That is, the relative abundance of *WPS-2* and *SAR324_cladeMarine_group_B* was significantly reduced in the yeast β-glucan group (*p* < 0.05) (Figure 4C).

### 3.8. Correlation between WPS-2 Abundance and SCFAs Concentration or Meat Drip-Loss-Related Biochemical Indices

**Figure 5:**
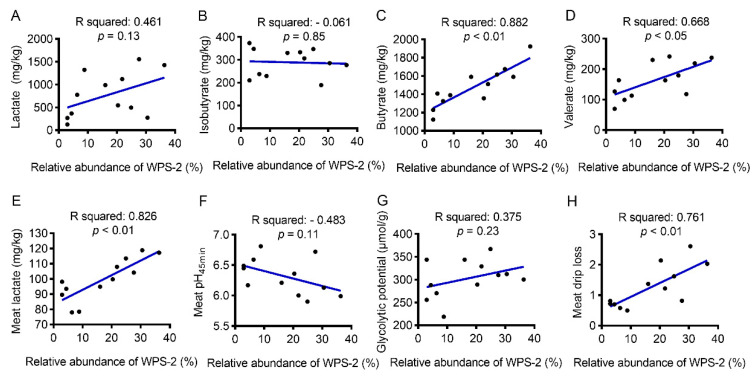
Correlation between the relative abundance of *WPS-2* in colonic digesta and SCFAs concentration or pork quality parameters. (**A**–**D**) Correlation between the relative abundance of *WPS-2* and SCFAs concentration in colonic digesta. (**E**–**H**) Correlation between the relative abundance of *WPS-2* and meat drip-loss-associated biochemical indices. Correlation analyses were performed using Pearson’s correlation coefficient (*n* = 6).

Considering the very limited proportion of *SAR324_cladeMarine_group_B* in the colonic digesta, we conducted a regression analysis between the relative abundance of *WPS-2* and SCFAs concentration to determine whether yeast-β-glucan-induced shifts in the production of SCFAs were linked to specific bacterial taxa in colonic digesta (Figure 5A–D). Strikingly, a significant linear relationship was detected between the *WPS-2* abundance and the concentration of butyrate (R^2^ = 0.882, *p* < 0.01) and valerate (R^2^ = 0.668, *p* < 0.05) (Figure 5C,D). A regression analysis was further performed to decipher the association between the relative abundance of *WPS-2* and meat drip-loss-associated biochemical indices (Figure 5E–H). Interestingly, the regression analysis revealed that lactate content (R^2^ = 0.826, *p* < 0.01) and drip loss (R^2^ = 0.761, *p* < 0.01) in the LT muscle had a strong positive correlation with the abundance of *WPS-2* in colonic digesta (Figure 5E,H).

## 4. Discussion

With the rapid development of the swine industry, more and more attention has been paid to the production of uniform and high-quality meat. The WHC is a key parameter that has an important impact on carcass yield, economic implications, nutritional value and eating quality of pork [39]. As reported by Aaslyng and Hviid [40], the coefficient of variance of the drip loss was higher than other meat quality traits in a Danish pig population and thus detrimental to the further processing. The negative correlation between drip loss and early postmortem pH values was also revealed in our previous study [41]. Considering the direct impact of diets on muscle characteristics [39], it is necessary to develop new dietary strategies to improve pork quality.

Antioxidants, such as grade seed proanthocyanidin, garcinol and resveratrol, have been added in diets to reduce pork drip loss through enhancing antioxidant capacity, decreasing postmortem glycolysis and changing muscle characteristics [42,43,44]. At present, the use of synthetic antioxidants is gradually decreasing due to consumer concerns over safety and toxicity [45]. Therefore, there have been great interests in natural antioxidants for husbandry animal production recently. β-glucan is a natural antioxidant widely present in yeast, mushrooms, bacteria, algae, barley and oat [46]. Previous studies have revealed the beneficial impact of yeast β-glucan on the growth performance of pigs through reducing gut oxidative stress and improving nutrient digestibility [21,47], but its impact on pork quality remains largely unknown. In this study, our data showed that some meat quality parameters were improved by dietary yeast β-glucan supplementation, such as drip loss, pH_45 min_ values and shear force. Particularly, a dietary supplementation of 200 mg/kg yeast β-glucan exhibited the highest values of pH_45 min_ and the lowest values of drip loss.

Glycolysis is a major metabolic process in the postmortem period and closely linked to many parameters of meat quality [48]. Under anaerobic conditions, the increase of glycolytic flux causes significant lactate and H^+^ accumulation, and there is a negative correlation between muscle ultimate pH and net lactate accumulation (R^2^ = 0.59) [49]. A greater positive correlation between drip loss and lactate content in the LT muscle was also observed in a previous study [50]. In the present work, dietary 200 mg/kg yeast β-glucan supplementation reduced drip loss and elevated pH_45 min_ values, paralleling a decrease in glycolytic potential and lactate content in the LT muscle, supporting that decreased glycolytic potential contributed to the improved meat quality after dietary yeast β-glucan treatment. Glycolytic potential is finely mediated by glycolytic enzymes. For instance, the activity of PGK1 in skeletal muscle was positively correlated with glycolytic potential, lactate content and glycogen content [51]. Additionally, LDH activity is regarded as a key anaerobic glycolytic index and reflects the lactate production in muscle to some extent [52]. Herein, dietary 200 mg/kg yeast β-glucan not only augmented the activity of PGK1 but also repressed the activity of LDH in the LT muscle, further corroborating the decreased glycolytic potential by yeast β-glucan. Furthermore, increasing the proportion of glycolytic fibers has been reported to induce the rapid postmortem muscle glycolysis [53]. To further understand the changes in the meat quality, *MyHC* isoforms expression in the LT muscle were examined in this study and results showed that dietary supplementation with 200 mg/kg yeast β-glucan only increased the mRNA level of *MyHC IIx*, representing the increased percentage of fast/oxidative-glycolytic fibers [54]. Accordingly, skeletal-muscle-fiber-type transformation was not potentially linked to the decreased glycolytic potential by yeast β-glucan supplementation. Nevertheless, the above results again strongly indicated the role of natural antioxidants in mitigating glycolysis.

It is well-known that ante-mortem stress accelerates the depletion of muscle ATP, which is responsible for the increased glycolytic potential [55,56]. Plasma cortisol plays an important role in stress-induced immunoreaction and ROS generation [57,58,59]. Indeed, studies have found that oxidative stress resulted in a significant increase of cortisol concentration in plasma [60], and plasma cortisol concentrations were positively correlated with the degrees of oxidative stress [61]. In addition, plasma ALB and GLB are important to improve body immunity and the decrease of the ratio of A/G increases the level of specific immune response [62]. Based on the antioxidant enzyme activities in muscle, decreased plasma cortisol content and increased plasma ALB as well as GLB content by yeast β-glucan treatment, we can deduce that yeast β-glucan supplementation decreased postmortem muscle glycolysis by increasing antioxidant capacity and suppressing preslaughter stress at least in part, thus contributing to the improved pork quality.

The gut microbiota–muscle axis is important in regulating muscle growth and metabolism through beneficial or harmful microbial metabolites produced by fermentation [63]. SCFAs, which are produced in the hindgut, not only act as signal molecules to impact intestinal health but also participate in the metabolism of peripheral organs [64]. In the current study, dietary supplementation of 200 mg/kg yeast β-glucan decreased the valerate content in colonic digesta and tended to reduce the content of lactate, isobutyrate and butyrate. Although dietary supplementation of 200 mg/kg yeast β-glucan had no effect on the α-diversity of microbiota, it should be noted that yeast β-glucan significantly decreased the *WPS-2* abundance as revealed by a Wilcoxon rank-sum test. *WPS-2* is an as-yet-uncultured bacterial clade [65], and its function is still mysterious. Strikingly, the abundance of *WPS-2* was positively correlated with drip loss and lactate concentration in skeletal muscle, and negatively linked to pH_45 min_, suggesting that the reduced abundance of *WPS-2* was potentially linked to the improved meat quality by yeast β-glucan. However, the direct contribution of *WPS-2* to muscle metabolism and characteristics is still largely unknown and merits further investigation.

Some limitations of this study should also be noted. Although we showed that 200 mg/kg yeast β-glucan supplementation reduced muscle glycolytic potential, the exact mechanism behind this beneficial effect is still largely unknown. Furthermore, the correlation between *WPS-2* abundance and some meat quality was established, however, the direct contribution of *WPS-2* to muscle metabolism and its characteristics is still unknown and merits further investigation. Fecal microbiota transplantation should also be conducted in the future to determine the role of gut microbiota in the improved pork quality by yeast β-glucan. Importantly, 100 mg/kg yeast β-glucan supplementation markedly reduced the fat-free lean index compared with control. The adverse effects of dietary yeast β-glucan supplementation on carcass traits should also not be ignored.

## 5. Conclusions

Here, we provided a novel finding that, as a natural antioxidant, yeast β-glucan supplementation in diets effectively improved the pH_45 min_ value and WHC of fresh pork, through reducing muscle postmortem glycolysis and increasing the antioxidant capacity of finishing pigs. The ingestion of diets containing yeast β-glucan decreased the relative abundance of phylum *WPS-2*, which was positively associated with the muscle lactate content and drip loss. Considering the reduced muscle glycolytic potential, increased antioxidant capacity and improved meat quality, dietary supplementation of 200 mg/kg yeast β-glucan is suggested in the diets of finishing pigs.

## Figures and Tables

**Table 1:** Ingredient composition and nutrient content of basal diet (%, as-fed basis).

Ingredient	Content, %	Nutrient Levels	Content
Corn	81.00	Analyzed nutrient levels	
Soybean meal	11.50	Crude protein	12.37
Wheat bran	3.20	Lysine	0.84
Soybean oil	1.20	Methionine + cysteine	0.44
L-Lysine·HCl, %	0.39	Threonine	0.53
DL-Methionine, 98.0%	0.03	Tryptophan	0.14
L-Threonine, 98.5%	0.11	Isoleucine	0.45
L-Tryptophan, %	0.03	Leucine	1.13
L-Valine, %	0.02	Valine	0.59
Limestone	0.70	Calculated nutrient levels	
Dicalcium phosphate	0.90	Digestible energy, MJ/kg	14.37
Salt	0.34	Metabolizable energy, MJ/kg	14.01
50% Choline chloride	0.08	Standardized ileal digestible amino acids	
Premix ^1^	0.50	Lysine	0.75
Total	100.00	Methionine + cysteine	0.42
		Threonine	0.46
		Tryptophan	0.13
		Isoleucine	0.40
		Leucine	1.03
		Valine	0.48

^1^ The premix provided the following per kilogram of diets: vitamin A, 6000 IU; vitamin D_3_, 2400 IU; vitamin E, 20 IU; vitamin K_3_, 2 mg; vitamin B_1_, 0.96 mg; vitamin B_2_, 4 mg; vitamin B_6_, 2 mg; vitamin B_12_, 0.012 mg; biotin, 0.04 mg; folic acid, 0.40 mg; pantothenic acid, 11.2 mg; nicotinic acid 22 mg; Cu, 120 mg; Fe, 76 mg; Mn, 12 mg; Zn, 76 mg; I, 0.24 mg; Se, 0.40 mg.

**Table 2:** Effects of dietary yeast β-glucan supplementation on immunological stress of finishing pigs (*n* = 8).

Items	Yeast β-Glucan Levels (mg/kg)					SEM	*p* Value		
	0	50	100	200	400		ANOVA	Linear	Quadratic
Albumin, g/L	32.50 ^b^	32.43 ^b^	33.61 ^ab^	34.95 ^a^	35.34 ^a^	0.63	<0.01	<0.01	0.05
Globulin, g/L	36.86 ^b^	39.22 ^ab^	39.75 ^ab^	41.30 ^a^	37.18 ^b^	1.15	0.05	0.83	<0.01
Totel protein, g/L	72.20	74.76	73.36	73.73	71.23	1.31	0.37	0.25	0.21
A/G	0.97 ^a^	0.90 ^ab^	0.86 ^ab^	0.79 ^b^	0.88 ^ab^	0.03	0.02	0.01	<0.01
Cortisol, ng/mL	145.10 ^a^	122.08 ^b^	127.10 ^ab^	122.17 ^b^	136.08 ^ab^	6.19	0.04	0.92	0.01

Note: values with different superscripts means significant difference (*p* < 0.05).

**Table 3:** Effects of dietary yeast β-glucan supplementation on carcass traits of finishing pigs (*n* = 8).

Items	Yeast β-Glucan Levels (mg/kg)					SEM	*p* Value		
	0	50	100	200	400		ANOVA	Linear	Quadratic
Live body weight, kg	110.11	112.28	111.16	110.44	110.37	1.53	0.86	0.70	0.80
Carcass weight, kg	83.37	84.23	82.61	82.00	81.08	0.93	0.17	0.02	0.79
Carcass length, cm	80.70	78.76	79.38	79.29	79.13	0.77	0.47	0.41	0.40
Dressing percentage, %	74.20	74.97	74.35	74.36	74.58	0.33	0.50	0.86	0.94
Back fat depth, mm									
Shoulder fat thickness	39.77	39.58	43.22	38.70	40.68	1.11	0.06	0.96	0.93
Last rib fat thickness	22.06	23.40	25.27	24.26	23.50	0.78	0.08	0.48	0.02
Lumbosacral fat thickness	17.49	17.27	18.10	16.94	16.59	0.73	0.65	0.27	0.77
6th to 7th rib fat thickness	28.27	28.74	30.99	26.46	28.25	1.36	0.24	0.52	0.84
10th rib fat thickness	20.33 ^ab^	20.12 ^b^	23.03 ^ab^	20.39 ^ab^	23.51 ^a^	0.79	0.01	0.01	0.67
Average back-fat depth	26.58	26.36	29.51	25.80	26.67	1.10	0.95	0.69	0.69
Loin eye area, cm^2^	43.56	45.24	42.24	39.78	42.24	1.88	0.36	0.30	0.21
Fat-free lean index, %	49.10 ^a^	48.77 ^ab^	47.71 ^b^	48.68 ^ab^	48.33 ^ab^	0.32	0.04	0.27	0.20

Note: values with different superscripts means significant difference (*p* < 0.05).

**Table 4:** Effects of dietary yeast β-glucan supplementation on meat quality and proximate analysis of the *Longissimus Thoracis* muscle in finishing pigs (*n* = 8).

Items	Yeast β-Glucan Levels (mg/kg)					SEM	*p* Value		
	0	50	100	200	400		ANOVA	Linear	Quadratic
Meat quality									
pH_45 min_	6.16 ^b^	6.23 ^b^	6.34 ^ab^	6.56 ^a^	6.42 ^ab^	0.07	<0.01	<0.01	<0.01
pH_24 h_	5.61	5.63	5.61	5.65	5.61	0.03	0.90	0.98	0.49
Flesh color score	1.69	1.84	1.80	2.07	1.93	0.11	0.15	0.10	0.09
L*_45 min_	44.28	42.50	42.61	41.01	40.81	1.03	0.14	0.02	0.25
a*_45 min_	14.01 ^ab^	14.12 ^ab^	13.83 ^b^	14.68 ^a^	14.66 ^a^	0.24	0.05	0.01	0.63
b*_45 min_	10.00	9.55	9.45	9.16	9.49	0.34	0.54	0.38	0.14
Drip loss, %	1.62 ^a^	1.27 ^ab^	1.33 ^ab^	0.86 ^b^	1.26 ^ab^	0.15	0.03	0.11	0.01
Cooking loss, %	26.75 ^ab^	26.55 ^ab^	30.20 ^a^	24.57 ^b^	24.53 ^b^	0.95	<0.01	0.01	0.50
Shear force, *n*	71.07 ^a^	60.74 ^b^	62.94 ^ab^	67.32 ^ab^	67.64 ^ab^	2.62	0.06	0.64	0.20
Marbling score	1.66	1.78	1.79	1.94	1.69	0.09	0.28	0.93	0.03
Proximate analysis									
Moisture, %	73.21	74.01	73.22	73.30	73.27	0.43	0.64	0.66	0.98
Crude protein, %	23.17	22.75	22.70	23.05	22.83	0.28	0.72	0.77	0.78
Intramuscular fat, %	4.00	3.50	3.88	4.05	3.69	0.24	0.48	0.80	0.58

Note: values with different superscripts means significant difference (*p* < 0.05).

## Data Availability

Data is contained within the article.

## Supplementary Materials

The following supporting information can be downloaded at: https://www.mdpi.com/article/10.3390/antiox11071340/s1. Table S1: Primers used for qRT-PCR, Table S2: Effects of dietary yeast β-glucan supplementation on texture profile analysis of the *Longissimus thoracis* muscle in finishing pigs (*n* = 8), Table S3: Effects of dietary yeast β-glucan supplementation on fatty acid composition of the *Longissimus thoracis* muscle in finishing pigs (% of fresh tissue, *n* = 8), Figure S1: Effects of dietary yeast β-glucan supplementation on mRNA expression of genes concerning myosin heavy-chain (*MyHC*) isoform of the *Longissimus thoracic* muscle in finishing pigs, Figure S2: Boxplots of alpha diversity as measured by diversity index of the colon microbiome.

## Abbreviations

WHC, water holding capacity; IMF, intramuscular fat; SCFAs, short-chain fatty acids; BW, body weight; LT, *longissimus thoracis*; ALB, albumin; TP, total protein; GLB, globulin; PKM1, pyruvate kinase M 1; LDH, lactate dehydrogenase; GAPDH, glyceraldehyde-3-phosphate dehydrogenase; PGK1, phosphoglycerate kinase 1; ROS, reactive oxygen species; MDA, malondialdehyde; CAT, catalase; SOD, superoxide dismutase; GSH-Px, glutathione peroxidase; T-AOC, total antioxidant capacity.
