# Supplementary material for: Effects of Dietary Yeast β-Glucan Supplementation on Meat Quality, Antioxidant Capacity and Gut Microbiota of Finishing Pigs
Source: Antioxidants (Basel). 2022 Jul 8;11(7):1340. doi: 10.3390/antiox11071340 (PMC9311933; doi:10.3390/antiox11071340)
Supplement: Supplementary file 1 [file antioxidants-11-01340-s001.zip › antioxidants-1781217-supplementary.pdf]

Supporting Information

Supplementary Tables

Table S1. Primers used for qRT-PCR

Table S2. Effects of dietary yeast β-glucan supplementation on texture profile analysis of the longissimus thoracis muscle in finishing pigs (n = 8).

Table S3. Effects of dietary yeast β-glucan supplementation on fatty acid composition of the longissimus thoracis muscle in finishing pigs (% of fresh tissue, n = 8)

Supplementary Figures

Figure S1. Effects of dietary yeast β-glucan supplementation on mRNA expression of genes concerning myosin heavy-chain (MyHC) isoform of the *M. longissimus thoracic* in finishing pigs.

Figure S2. Boxplots of alpha diversity as measured by diversity index of the colon microbiome.

Table S1. Primers used for qRT-PCR

Gene	Primer Sequence (5' to 3')	GeneBank No.	TM (℃)
GAPDH	F: TCGGAGTGAACGGATTTG<br>R: CCTGGAAGATGGTGATGG	NM_001206359.1	60
LDH	F: AGTCGTCTCAATCTGGTGCA<br>R: CACACGGTGCTTGGGTAATC	NM_001113287.1	62
PGK1	F: CGCCACTGAATTGCCAAGAT<br>R: CATGACGACCCTCTTTCCCT	NM_001099932.2	58
MyHC I	F: CGTGGACTACAACATCATAGGC<br>R: CTTTGCCCTTCTCAACAGGT	NM_213855	64
MyHC IIa	F: GGAGATCGACGACCTTGCTA<br>R: CTCCTTGGATTTCAGCTCGC	NM_214136	58
MyHC IIx	F: GAAACCGTCAAGGGTCTACG<br>R: CGCTTCCTCAGCTTGTCTCT	NM_001104951	64
MyHC IIb	F: GTTCTGAAGAGGGTGGTAC<br>R: AGATGCGGATGCCCTCCA	NM_001123141	63
SOD1	F: AGACCTGGGCAATGTGACTG<br>R: GTGCGGCCAATGATGGAATG	NM_001190422	62
GPX1	F: GTGAATGGCGCAAATGCTCA<br>R: ATTGCGACACACTGGAGACC	NM_214201	60
GST	F: CCAACCCAGAAGACTGCTCA<br>R: CATTCAGGTGGGCTCTTCGT	AB000884	60
Keap1	F: ACGACGTGGAGACAGAAACGT<br>R: GCTTCGCCGATGCTTCA	NM_001114671	62
NRF2	F: GCCCCTGGAAGCGTTAAAC<br>R: GGACTGTATCCCCAGAAGGTTGT	XM_003133500	60
CAT	F: CAGATGAAGCATTGGAAGGAGC<br>R: TTGTCTCCTATCGGATTCCCAG	NM_214301	60
18sRNA	F: GAGCGAAAGCATTTGCCAAG<br>R: GGCATCGTTTATGGTCGGAAC	NM_001244062	60

Table S2. Effects of dietary yeast β-glucan supplementation on texture profile analysis of the Longissimus Thoracis muscle in finishing pigs (n = 8)

**Items**	**Yeast β-glucan levels (mg/kg)**					**SEM**	***P* value**		
	**0**	**50**	**100**	**200**	**400**		**ANOVA**	**Linear**	**Quadratic**
Hardness, N	39.34	32.42	38.61	35.08	35.45	2.34	0.24	0.54	0.61
Adhesiveness, mJ	0.07	0.08	0.09	0.08	0.07	0.01	0.57	0.75	0.19
Cohesiveness, Ratio	0.45	0.44	0.48	0.48	0.46	0.01	0.02	0.28	0.03
Springiness, mm	2.99	3.04	3.09	3.07	2.99	0.08	0.82	0.82	0.27
Gumminess, N	19.02^{ab}	15.59^{b}	22.70^{a}	18.65^{ab}	18.09^{ab}	1.49	0.03	0.85	0.33
Chewiness, mJ	50.89	48.82	63.05	55.32	54.74	4.47	0.23	0.58	0.23

Note: Values with different superscripts means significant difference ($p < 0.05$).

Table S3. Effects of dietary yeast β-glucan supplementation on fatty acid composition of the Longissimus Thoracis muscle in finishing pigs (% of Fresh Tissue, n = 8)

Items	Yeast β-glucan levels (mg/kg)					SEM	*P* value		
	0	50	100	200	400		ANOVA	Linear	Quadratic
C14:0	1.20	1.38	1.37	1.39	1.40	0.08	0.36	0.19	0.24
C16:0	25.99	26.23	26.53	26.29	26.76	0.29	0.41	0.10	0.86
C16:1	3.23	3.25	3.02	3.22	3.41	0.12	0.26	0.16	0.20
C17:0	0.17	0.20	0.19	0.18	0.18	0.01	0.54	0.83	0.53
C18:0	14.19	14.48	14.97	14.96	14.46	0.30	0.28	0.70	0.04
C18:1n9c	41.97	41.56	42.00	41.26	41.10	0.52	0.64	0.20	0.87
C18:2n6c	8.43	8.79	8.00	8.47	8.47	0.55	0.90	0.99	0.80
C18:3n3	0.31	0.31	0.31	0.30	0.32	0.01	0.93	0.72	0.65
C20:0	0.27	0.30	0.28	0.28	0.30	0.01	0.41	0.37	0.85
C20:1	0.75	0.77	0.72	0.75	0.73	0.03	0.88	0.66	0.83
C21:0	0.35	0.38	0.36	0.37	0.37	0.02	0.75	0.52	0.84
C20:3n6	0.20	0.23	0.18	0.21	0.24	0.02	0.28	0.23	0.50
C20:4n6	1.39	1.61	1.25	1.55	1.70	0.17	0.39	0.21	0.67
C20:3n3	0.04	0.06	0.05	0.06	0.07	0.01	0.42	0.13	0.79
C24:0	0.04	0.05	0.04	0.05	0.05	< 0.01	0.32	0.08	0.90
SFA	42.74	42.87	44.27	43.84	44.09	0.53	0.16	0.08	0.21
MUFA	46.13	45.69	44.54	45.35	45.42	0.62	0.49	0.64	0.25
PUFA	10.58	11.26	10.88	10.81	11.03	0.83	0.98	0.88	0.96
n-3 PUFA	0.49	0.57	0.51	0.51	0.53	0.04	0.65	0.88	1.00
n-6 PUFA	10.01	10.63	9.44	10.23	10.40	0.72	0.81	0.75	0.75
Total fatty acids	82.41	88.72	89.60	73.47	71.87	10.47	0.65	0.23	0.95

.

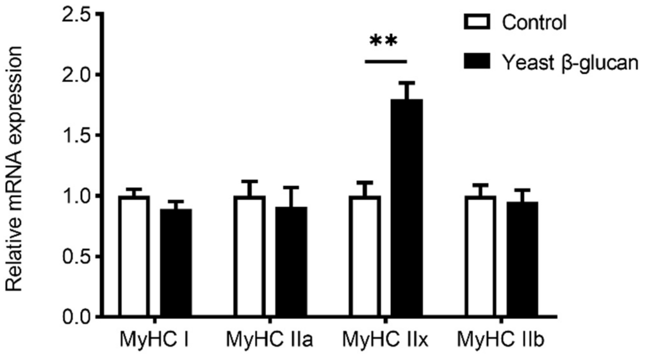

Figure S1. Effects of dietary yeast β-glucan supplementation on mRNA expression of genes concerning myosin heavy-chain (MyHC) isoform of the M. longissimus thoracic in finishing pigs. Yeast β-glucan, dietary supplementation of 200 mg/kg yeast β-glucan. Data are expressed as the means ± SEMs (n = 8). ** $p < 0.01$.

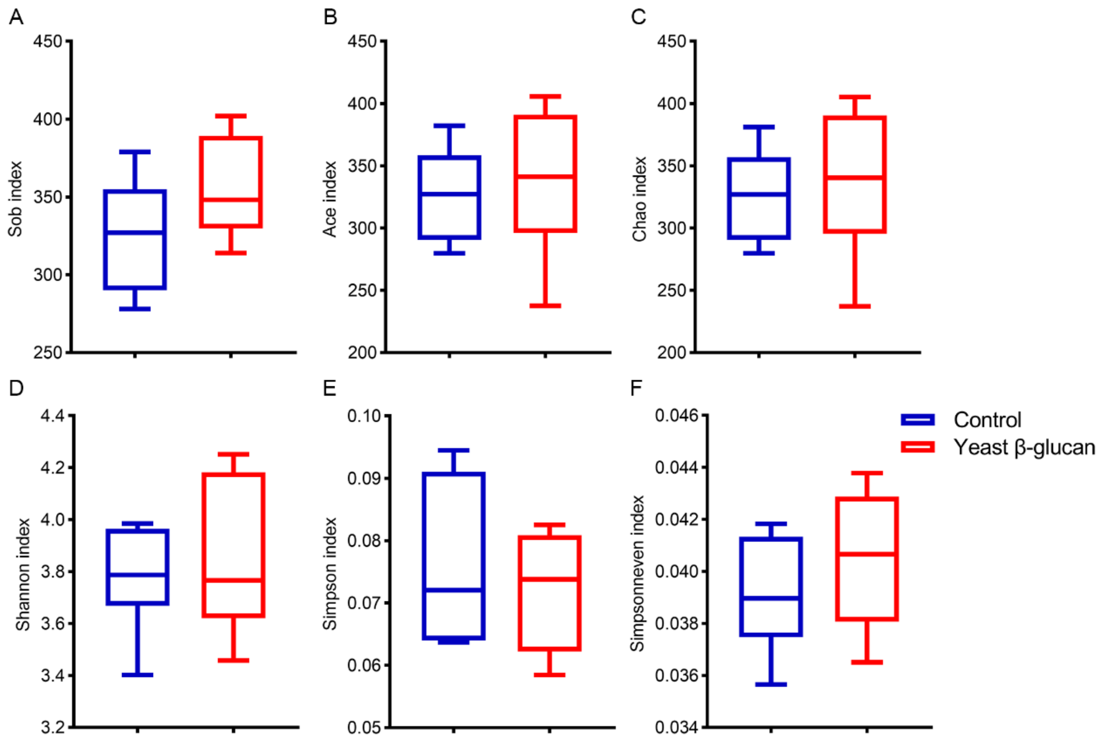

Figure S2. Boxplots of alpha diversity as measured by diversity index of the colon microbiome. Diversity index of (A) sob index, (B) Ace, (C) Chao, (D) Shannon, (E) Simpson and (F) Simpsonneven. Yeast β-glucan, dietary supplementation of 200 mg/kg yeast β-glucan. Alpha diversity indices were analyzed in Kruskal-Wallis to test for differences (n = 6).
